# Childbirth experience questionnaire (CEQ): development and evaluation of a multidimensional instrument

**DOI:** 10.1186/1471-2393-10-81

**Published:** 2010-12-10

**Authors:** Anna Dencker, Charles Taft, Liselotte Bergqvist, Håkan Lilja, Marie Berg

**Affiliations:** 1 Institute of Health and Care Sciences, Sahlgrenska Academy, University of Gothenburg, Gothenburg, Sweden; 2 Department of Obstetrics and Gynecology, Perinatal Center, Sahlgrenska University Hospital, Gothenburg, Sweden

## Abstract

**Background:**

Negative experiences of first childbirth increase risks for maternal postpartum depression and may negatively affect mothers' attitudes toward future pregnancies and choice of delivery method. Postpartum questionnaires assessing mothers' childbirth experiences are needed to aid in identifying mothers in need of support and counselling and in isolating areas of labour and birth management and care potentially in need of improvement. The aim of this study was to develop and evaluate a questionnaire for assessing different aspects of first-time mothers' childbirth experiences.

**Methods:**

Childbirth domains were derived from literature searches, discussions with experienced midwives and interviews with first-time mothers. A draft version of the Childbirth Experience Questionnaire (CEQ) was pilot tested for face validity among 25 primiparous women. The revised questionnaire was mailed one month postpartum to 1177 primiparous women with a normal pregnancy and spontaneous onset of active labor and 920 returned evaluable questionnaires. Exploratory factor analysis using principal components analysis and promax rotation was performed to identify dimensions of the childbirth experience. Multitrait scaling analysis was performed to test scaling assumptions and reliability of scales. Discriminant validity was assessed by comparing scores from subgroups known to differ in childbirth experiences.

**Results:**

Factor analysis of the 22 item questionnaire yielded four factors accounting for 54% of the variance. The dimensions were labelled *Own capacity, Professional support, Perceived safety*, and *Participation*. Multitrait scaling analysis confirmed the fit of the four-dimensional model and scaling success was achieved in all four sub-scales. The questionnaire showed good sensitivity with dimensions discriminating well between groups hypothesized to differ in experience of childbirth.

**Conclusion:**

The CEQ measures important dimensions of the first childbirth experience and may be used to measure different aspects of maternal satisfaction with labour and birth.

## Background

Childbirth is described as a multifaceted experience. Sense of security and perceived control, experienced level of labour pain, personal support, midwifery care, experience of earlier deliveries, intrapartum analgesia, information given and involvement in decision-making contribute to the childbirth experience [1-9]. Unplanned medical interventions during childbirth, e.g. oxytocin augmentation, emergency caesarean and operative vaginal deliveries, intrapartum complications and need of neonatal intensive care are related to maternal dissatisfaction [8-13].

First-time mothers are particularly vulnerable for negative experiences and in Sweden approximately 9% of all primiparous women are dissatisfied with the birth experience one year after the birth [13]. Negative experiences increase the risk for maternal postpartum depression and may negatively affect attitudes to future pregnancies and births and may prompt women to request caesarean delivery [14]. Therefore, knowledge of factors affecting maternal satisfaction is important in order to improve childbirth care. For this purpose, postpartum questionnaires covering multidimensional aspects are needed to comprehensively explore women's experiences of childbirth.

Existing instruments for assessing maternal experiences during labour and birth [15-18] either assess isolated aspects of the childbirth experience or aggregate diverse aspects into single overall scores. For example, the Labour Agentry Scale (LAS) [15,19] covers only one childbirth dimension, i.e. perceived personal control during pregnancy and childbirth. Another questionnaire used by Lavender et al. [16] aggregates ratings of different aspects of childbirth experience to a single score of maternal experience. Likewise, the Wijma Delivery Expectancy/Experience Questionnaire (W-DEQ) [17] measures fear of childbirth in one domain. The Childbirth Self-efficacy Inventory (CBSEI) is also a unidimensional instrument measuring self-efficacy and coping during childbirth [18]. An exception is the Quality from the Patient's Perspective Intrapartal (QPP-I) instrument which assesses women's perceptions of childbirth care [20] in both one general factor and 10 subfactors. However, this questionnaire was developed primarily for evaluating quality of care and many of the domains assess aspects of the physical environment or the performance of different healthcare professionals. Hence, validated postpartum questionnaires covering multidimensional aspects that comprehensively explore women's perceptions of and feelings about their first labour and birth are lacking.

The aim of this study was therefore to develop and evaluate a questionnaire measuring different dimensions of childbirth experience in first time mothers.

## Methods

### Instrument development

The Childbirth Experience Questionnaire (CEQ) was developed to study women's perceptions of first labour and birth. Pertinent domains were identified through literature studies, discussions in a group of four experienced midwives and one senior obstetrician and a group interview one month postpartum with twelve mothers, most of them primiparous. Identified areas of childbirth experience included intrapartum sense of security, experience of labour pain, partner's support, midwifery care and support, memories from the childbirth and experience of own performance. The items regarding memories came from the women's narratives one month postpartum. Items were drafted to cover the identified areas and formulated as positive and negative statements. The response format was a 4-point Likert scale ranging from 1 (Totally agree), 2 (Mostly agree), 3 (Mostly disagree) to 4 (Totally disagree). Memory of labour pain, sense of security and control were assessed with visual analogue scales (VAS). A draft version of the CEQ was pilot tested for face validity (i.e. comprehension and relevance) in 25 primiparous women. Based on the women's comments, the questionnaire was revised and comprised totally 28 items. A one-month follow-up frame was chosen. At that point the mothers were expected to have gone through the first relief phase but still have relatively fresh memories of the childbirth experience. Moreover, the women had little or no contact with childbirth caregivers after one month and were therefore less likely to give socially desirable responses.

### Study sample

The sample included 1177 women who participated in a study on labour progress and oxytocin augmentation between October 1998 and December 2003 at Sahlgrenska University Hospital in Gothenburg and Ryhov County Hospital in Jönköping, Sweden. The study is described in detail elsewhere [21]. In brief, the antenatal clinics provided written information about the study as well as information about the follow-up questionnaire to healthy nulliparous women in their third trimester. Inclusion criteria were nulliparity, a singleton fetus in cephalic presentation, uncomplicated pregnancy, spontaneous onset of active labor with regular contractions and an effaced cervix dilated not less than four centimeters and gestational age of between 37+0 and 41+6 weeks at admission to the delivery ward. Informed consent was obtained from all respondents who were mailed the questionnaire one month postpartum.

### Statistical and psychometric analyses

Descriptive statistics were computed to characterize item score distributions. Item response completeness and frequency distributions were examined to identify items with high missing data rates and/or high ceiling/floor effects. The VAS-scales scores were transformed to categorical values, 0-40 = 1, 41-60 = 2, 61-80 = 3 and 81-100 = 4. Ratings of positively worded statements and the pain items were reversed so that higher scores reflect more positive scoring.

Exploratory factor analyses using principal components analysis were performed to examine the construct validity of the CEQ. Both orthogonal (varimax) and oblique (promax) rotations were conducted. Oblique rotation was selected due to the fact that the dimensions were correlated (r = 0.23-0.63). Items with extreme ceiling effects were not entered in the analysis. The Kaiser rule (eigenvalue > 1.0) was applied for determining the number of dimensions to extract. Factor extraction was also guided by the clinical interpretability of the factors. Items with maximum loadings less than 0.40 were dropped.

Multitrait scaling analysis [22] was performed in order to confirm the derived factor structure and to test scaling assumptions for aggregating item ratings of the dimensions. Four assumptions were tested: item internal consistency (item-hypothesized scale correlations ≥ 0.40 and Cronbach's alpha ≥ 0.70), item discriminant validity (item-hypothesized scale correlation > item-other scale), equal item-hypothesized scale correlation (item-scale correlations roughly the same for all items in scale), item equal variance (variances of items in hypothesized scale roughly equal).

Item ratings were aggregated to scale scores for each respondent using the half scale method, i.e., mean values were computed when the respondent had answered at least half of the items in the scale [23].

The method of known-groups validation [24] was used to assess the ability of the CEQ to distinguish between subgroups known to differ on key sociodemographic or clinical variables. Based on previous research results [8-13], it was hypothesized that the women who had a labour lasting more than twelve hours, women who had the labour augmented with oxytocin and women who had experienced an operative delivery would score lower on all scales. Scale scores were compared between groups with Mann-Whitney U-test. All significance tests were two-tailed and conducted at the 5 percent significance level. The Holm-Bonferroni correction method was used when multiple tests were performed [25]. Accordingly, the p-values were ranked from lowest to highest and the smallest p-value was compared to 0.05 divided by 4 (the total number of tests), the second smallest with 0.05 divided by 3 (the total number of tests minus one) and so forth. Effect sizes, as defined by Cohen [26], were computed as the difference between group mean scores divided by the pooled standard deviation of the two groups. In line with criteria suggested by Cohen, effect sizes of 0.2-0.5 was regarded as "small", 0.5-0.8 as "moderate" and above 0.8 as "large" [23].

All statistical analyses were performed using SPSS 15.0 for Windows (SPSS Inc., Chicago, IL, USA) with the exception of multitrait scaling analysis for which the MAP-R program was used [22].

## Ethical approval

Permission to conduct the study was obtained from the Regional Ethics Board in Gothenburg in November 1997 (L586-97).

## Results

A total of 920 primiparous women (78% response rate) returned evaluable questionnaires. Characteristics of the study population are shown in Table 1. Twenty-six percent of the women had a labour lasting more than 12 hours and operative delivery accounted for 17% of the births (12% instrumental vaginal deliveries and 5% emergency caesareans).

**Table 1:** Characteristics of study population, n = 920.

Age years, mean	28.1 (4.4)
Gestational age weeks, mean	40.2 (1.0)
Spontaneous vaginal delivery	764 (83.0%)
Operative delivery:	156 (17.0%)
*Instrumental vaginal delivery*	109 (11.8%)
*Caesarean delivery*	47 (5.1%)
Labour duration more than 12 hours	236 (25.7%)
Oxytocin augmentation during labour	617 (67.1%)
Postpartum haemorrhage mL	490 (359)
Sphincter laceration/vaginal births	39 (4.5%)
Apgar score < 7 at 5 minutes	9 (1.0%)

Data are given as mean (SD) or as n (%).

### Item rating distributions

Item rating distributions were first examined for completeness and skewness. Missing value rates were low for all items. Two items regarding the partner's support and one item about the baby *(My assurance that the baby was doing well made me feel secure) *showed extreme ceiling effects (80-90% endorsed the most positive response choice) and were excluded from further analysis. Six items concerning midwifery support and care, including one about the medical competence also had high ceiling effects (60%); however, these items were considered to be of clinical importance and were hence retained.

### Factor analysis

In total, 25 items were initially entered in the exploratory factor analysis. Two items with maximum loadings less than 0.40 on any domain were excluded: *"I have memory lapses from the labour process" *and *"Experienced pain in second stage"*. The item "*The care given by my midwife made me feel secure" *lowered Cronbach's alpha for its factor and was also excluded. After eliminating these 3 items, four dimensions met extraction criteria and were retained: O*wn capacity *(8 items regarding sense of control, personal feelings during childbirth and labour pain), *Professional support *(5 items about information and midwifery care), *Perceived safety *(6 items regarding sense of security and memories from the childbirth), and *Participation *(3 items regarding own possibilities to influence the birthing situation). The four factors accounted for 28, 15, 6, and 5% of the variance, respectively, for a total of 54% (Table 2).

**Table 2:** Matrix with factor loadings from exploratory factor analysis and principal component analysis with promax rotation.

Item	Own capacity	Professional support	Perceived safety	Participation
Experienced level of labour pain in dilatation stage, VAS^# ¤^	0.73		-0.40	
I felt strong ^§^	0.73			
I felt capable ^§^	0.73			
Experienced level of control, VAS^# ∂^	0.62			
I felt happy ^§^	0.61			
I felt that I handled the situation well	0.55			
I felt tired ^§^	0.53			
The labour progress went as I had expected	0.47			
My midwife also devoted enough time to my partner		0.82		
I felt very well taken care of by the midwife		0.87		
My midwife devoted enough time to me		0.86		
My midwife kept me informed about what was happening during labour and birth		0.80		
My midwife understood my needs		0.76		
Experienced level of sense of security, VAS^# ¥^			0.80	
Some of my memories from the labour process make me feel depressed			0.73	
My impression of the medical competence made me feel secure			0.59	
I have many negative memories from the labour process			0.59	
I have many positive memories from the labour process			0.56	
I felt scared ^§^			0.51	
I felt I could choose whether I should be up and moving or lie down				0.84
I felt I could choose the delivery position				0.80
I felt I could choose which pain relief method to use				0.58
Eigenvalue	6.1	3.3	1.4	1.1
Variance explained	27.8	14.8	6.5	5.1
Cumulative variance explained	27.8	42.6	49.1	54.3

Factor loadings < 0.30 are not shown.

Excluded items were: *The presence of my partner made me feel secure, I felt that my partner managed the situation well, My assurance that the baby was doing well made me feel secure, Experienced level of labour pain in expulsion stage VAS, The care given by my midwife made me feel secure *and *I have memory lapses from the labour process*.

^# ^In summary. The VAS-scales scores were transformed to categorical values, 0-40 = 1, 41-60 = 2, 61-80 = 3 and 81-100 = 4. The pain scores were reversed.

^§ ^...during labour and birth.

^¤ ^Anchor values: No pain-Worst imaginable pain.

^∂ ^Anchor values: No control-Complete control.

^¥ ^Anchor values: No sense of security-Feel totally secure.

### Multitrait scaling analysis

The multitrait scaling analysis showed that scaling assumptions were adequately met for all dimensions (Table 3). Item-scale correlations exceeded 0.40 for nearly all items and those less than 0.40 were still higher with their own scale than with competing scales (item discriminant validity). Cronbach's alpha coefficients were acceptable for group analyses (> 0.70) in all but the participation scale. Item-scale correlations, means and variances showed that items contributed roughly equally to its hypothesized scale.

**Table 3:** Tests of scaling assumptions.

Scale	Item internal consistency		Item discriminant validity^¤^	Item-scale correlation, r_p _range^∂^	Item means & variance, M (SD) range^¥^
				
	Item-scale correlations^# ^	Cronbach's alpha^§ ^			
Own capacity	7/8	0.82	8/8	0.30-0.69	1.89-3.25 (0.76-1.03)
Professional support	5/5	0.88	5/5	0.69-0.74	3.64-3.79 (0.52-0.67)
Perceived safety	5/6	0.78	6/6	0.36-0.67	2.99-3.69 (0.52-1.01)
Participation	2/3	0.62	3/3	0.33-0.56	3.35-3.71 (0.60-0.90)

Scaling assumptions:

^# ^Item-hypothesized scale correlations ≥ 0.40, corrected for overlap/number of correlations.

^§ ^Scale internal consistency reliability coefficients (Cronbach's alpha).

^¤ ^Item-hypothesized scale correlation significantly higher or higher than item-other scale.

^∂ ^Item-scale correlations roughly the same for all items in scale.

^¥ ^Items in hypothesized scale have roughly equal means and variances.

### Known-groups validation

**Figure 1:**
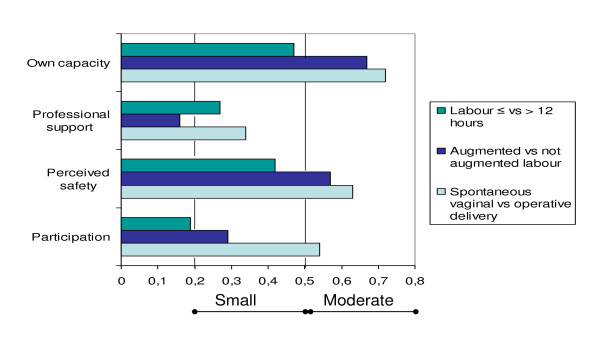
**Effect sizes between groups known to differ in childbirth experiences**. 0.2-0.5 = Small, 0.5-0.8 = Moderate, > 0.8 = Large.

Sensitivity of the model was examined by comparing mean scores on each subscale between women with labour duration more versus less than 12 hours; women with versus without oxytocin augmentation during labour; and women who had a spontaneous vaginal delivery versus those who had an operative delivery (emergency caesarean or instrumental vaginal birth). Women with prolonged labour scored lower in all scales, see Table 4. Effect sizes were, however, small (*Own capacity*, 0.47; *Perceived safety*, 0.42 and *Professional support*, 0.27) to trivial (*Participation*, 0.19). Women with oxytocin treatment scored significantly lower on all subscales. Effect sizes were moderate (*Own capacity, 0.67; Perceived safety, 0.57*), small (*Participation, 0.29) *or trivial (*Professional support, 0.16*). Women with operative births scored significantly lower on all four subscales, see Table 4. Effect sizes were moderate (*Own capacity*, 0.72; *Perceived safety*, 0.63; *Participation*, 0.54) or small (*Professional support*, 0.34), see Figure 1.

**Table 4:** Disciminant validity: differences in subscale scores between groups.

	Own capacity	Professional support	Perceived safety	Participation
Labour duration ≤ 12 hours, n = 684	2.68 (0.57)	3.73 (0.47)	3.37 (0.55)	3.61 (0.54)
Labour duration > 12 hours, n = 236	2.40 (0.60)	3.59 (0.63)	3.13 (0.64)	3.51 (0.55)
Unadjusted p-value ^#^	< 0.001	0.008	< 0.001	0.004
Holm-Bonferroni adjusted p-value ^§#^	0.0125	0.05	0.017	0.025
Effect size ^#^	0.47	0.27	0.42	0.19

No oxytocin augmentation, n = 303	2.88 (0.53)	3.75 (0.44)	3.53 (0.47)	3.69 (0.47)
Oxytocin augmentation during labour, n = 617	2.48 (0.58)	3.67 (0.55)	3.20 (0.60)	3.53 (0.57)
Unadjusted p-value ^¤^	< 0.001	0.037	< 0.001	< 0.001
Holm-Bonferroni adjusted p-value ^§¤^	0.0125	0.05	0.017	0.025
Effect size ^¤^	0.67	0.16	0.57	0.29

Spontaneous vaginal delivery, n = 764	2.67 (0.57)	3.72 (0.47)	3.37 (0.55)	3.63 (0.52)
Operative delivery ^∂^, n = 156	2.25 (0.58)	3.55 (0.69)	3.00 (0.65)	3.33 (0.64)
Unadjusted p-value ^¥^	< 0.001	0.022	< 0.001	< 0.001
Holm-Bonferroni adjusted p-value ^§¥^	0.0125	0.05	0.017	0.025
Effect size ^¥^	0.72	0.34	0.63	0.54

Data are given as mean (SD). Mann-Whitney U-test is used to compute p-values and Holm-Bonferroni correction was used.

^# ^Labour duration ≤ 12 vs > 12 hours.

^§ ^p-value for significance.

^¤ ^Oxytocin augmentation during labour vs no augmentation.

^∂ ^Caesarean and instrumental vaginal births.

^¥ ^Spontaneous vaginal vs Operative birth.

## Discussion

Factor analysis of the 22 item Childbirth Experience Questionnaire (CEQ) yielded four major dimensions of the childbirth experience, namely *Own capacity, Perceived safety, Professional support *and *Participation*

These domains confirmed the areas of the childbirth experience found in our literature searches [4-9] and the interviews with mothers one month postpartum. The psychometric properties of the CEQ were also found to be good. Specifically, scaling success was achieved in all four sub-scales and the instrument discriminated well between groups hypothesized to differ in experience of childbirth.

The dimension that explained most of the variance, *Own capacity*, included items regarding experienced emotions and sense of control, together with experienced labour pain. This dimension corresponds with the Labour Agentry Scale [15], measuring perceived personal control; however, our factor also included an item tapping perceived pain. Labour pain is considered to be a very significant part of childbirth [9,27] and experienced pain is likely to be strongly associated with the mother's sense of control in childbirth. *Own capacity *also has a component of self-efficacy (*I felt capable*) and coping (*I felt that I handled the situation well*) and thus likens the domain-specific Childbirth Self-efficacy Inventory scale [18]. Unlike the above instruments, the recently validated QPP-I measures 10 domains of intrapartal care [20]. Developed primarily for use in evaluating quality of care, many of the domains assess aspects of the physical environment or the role performance of different professionals. Although these aspects are of importance from a quality improvement perspective, our instrument focuses on the mother's experiences of her own performance and sense of support. There are obvious tradeoffs between domain-specific measures, such as the Labour Agentry Scale, and more generic measures, such as ours, that aim to comprehensively assess the childbirth experience. In future studies we plan to conduct head-on-head comparisons to examine the benefits and weaknesses of our questionnaire in relation to established domain-specific questionnaires.

Items regarding sense of security correlated with statements about memories and formed the dimension labelled *Perceived safety*. A possible explanation for the intercorrelations between fear, sense of security and memories from the childbirth is that distressing recollections are symptoms after experienced fear [28]. Childbirth is a stressful event and some women have traumatic stress symptoms, e.g. anxiety and fear of childbirth postpartum [29-31]. It has also been shown that feelings of safety during childbirth influence the memories from the birth [32]. This is an important area and should be included in an overall assessment of the childbirth experience. In contrast, the W-DEQ-scale specifically measures fear of childbirth with questions about anxiety, control and personal feelings in one domain [17] and does not include items regarding memory. Instead all questions are asked both before and after the birth, which is a way to include the influence on the memory. In another instrument [33] fear related to childbirth, in both men and women, is measured with 18 items about fear in four factors, all related to fear. One of the factors is about insecurity and danger, e.g. "Childbirth is a very risky situation". The safety dimension in our questionnaire includes items not unlike these items but most of them are formulated as positive statements, e.g. "*My impression of the medical competence made me feel secure*". Adding more negatively formulated items can possibly further enhance the discriminatory qualities in this scale.

As mentioned above, three items were found to have very high ceiling effects and were therefore not entered into the factor analysis. These items covered two separate domains, namely partner support and maternal worries about the baby. Although these domains have been identified as highly important aspects of the childbirth experience, we reasoned that they would be of little discriminative value [23]. The item *Memory lapses from the childbirth *did not fit into the factor model but may possibly contribute to the knowledge of this phenomenon. Memory lapses after labour and birth have been reported earlier [34] but this aspect of the birth experience is not well studied.

The strengths of this study are that domains and items comprising the CEQ were derived from patient interviews, discussions among experienced midwives and other staff, and pilot tested for face validity in a group of primiparous women. The psychometric performance of the questionnaire was tested on a large and representative sample of primiparous women with spontaneous onset of active labour (n = 920) living in various settings in Sweden. This heterogeneous sample represents a large group of childbearing women, as approximately 70% of all women expecting their first child are healthy, have a normal pregnancy and experience a spontaneous start of active labour.

The CEQ discriminated between groups of women known to differ in childbirth experiences [8-13]. Specifically, women with labour lasting more than 12 hours had, as expected, significantly lower scores on all scales than did those with shorter labour. Women who had their labour augmented scored lower than those without the treatment. Likewise, women who had delivered operatively had significantly lower scores than did those delivering normally (Table 4). On the other hand, effect sizes were primarily moderate to small, suggesting that further efforts may need to be made to improve the sensitivity of the questionnaire, especially regarding *Professional support *and *Participation*, where particularly weak effect sizes were observed (Figure 1).

The discriminative ability of the *Participation *scale may possibly have been debilitated by the fact that it comprised relatively few items (3 items) and that these items had comparatively poor internal consistency, as reflected by a low Cronbach's alpha (0.62). Adding relevant items and rewording existing items may help to augment the sensitivity of this scale. A reason for the weaker performance of the *Professional support *scale may be that the distribution of scores was skewed, with substantial ceiling effects. This problem is common in other scales and instruments measuring patient satisfaction with care and has serious implications for their ability to detect differences both between groups and over time [35]. A possible solution to this problem may be to extend the response scale from a four to a 5-step scale. For example, Moret et al. [36] have shown that extending the number of response alternatives substantially reduces or eliminates ceiling effects [37].

## Conclusions

In summary, this study reports on the development and testing of the Childbirth Experience Questionnaire to assess different aspects of women's experiences of first childbirth. The four dimensions revealed in the exploratory factor analysis are concordant with earlier research and cover important aspects of the childbirth experience [1-9]. Furthermore, the instrument discriminates well between groups known to differ in childbirth experiences. A normal first childbirth with a positive experience is very important with regard to future pregnancies and childbirths. Women with a negative experience and a severe fear of childbirth often request an elective caesarean delivery [14] which is associated with increased risks for both mother and baby [38-42]. Postpartum questionnaires are needed 1.) to explore how different types of labour management influence the women's childbirth experiences, 2.) to improve care during childbirth and 3.) to identify those in need of support and counselling after a negative birth experience. Although most current instruments assess single domains of the childbirth experience, there is also a need for questionnaires that reflect and comprehensively assess the multidimensional nature of women's experiences of childbirth. We suggest that this four-dimensional model adequately reflects the multidimensionality of the childbirth experience and can be used as a tool to identify women with negative experiences and for evaluating efforts to improve the quality of childbirth care.

## Competing interests

The authors declare that they have no competing interests.

## Authors' contributions

AD, LB and HL conducted the study. AD, CT, LB, HL and MB analyzed the data. AD wrote the paper and prepared the figures and tables. All the authors revised the paper and agreed to the submission of the final version of the manuscript.

## Pre-publication history

The pre-publication history for this paper can be accessed here:

http://www.biomedcentral.com/1471-2393/10/81/prepub
